# Production of protein hydrolysate containing antioxidant activity from *Hermetia illucens*

**DOI:** 10.1016/j.heliyon.2019.e02005

**Published:** 2019-06-28

**Authors:** Mochamad Firmansyah, Muhammad Yusuf Abduh

**Affiliations:** a School of Life Sciences and Technology, Institut Teknologi Bandung, Jalan Ganesha 10, 40132 Bandung, Indonesia; b Bioscience and Biotechnology Research Center, Institut Teknologi Bandung, Jalan Ganesha 10, 40132 Bandung, Indonesia

**Keywords:** Biochemistry, Biotechnology, Chemical engineering, Black soldier fly larvae, Enzymatic hydrolysis, Protein hydrolysate, Amino acid, Antioxidant activity

## Abstract

Protein hydrolysate is a complex mixture of peptide and amino acids that can be produced from various biomass sources including insects, such as black soldier fly larvae (*Hermetia illicens*) due to its relatively high protein content. This study aimed to investigate the potential of protein hydrolysate from black soldier fly larvae as a bioactive hydrolysate through enzymatic hydrolysis using bromelain. Black soldier fly larvae contain 25.6% protein and 35.5% lipids as determined by a proximate analysis. Experiments for the enzymatic hydrolysis of black soldier fly larvae was designed using a central composite design with three factors particularly enzyme concentration (1–5%), pH (6–8) and time of hydrolysis (3–24 hours). The protein hydrolysate had a yield of 10.70 % (on a weight basis) based on defatted biomass with a productivity of 21 mg/L/batch. The protein concentration varied between 240-310 μg/ml with the degree of hydrolysis varied in the range of 10–43%. The protein hydrolysate had a molecular weight in the range of 14–25 kDa based on Sodium Dodecyl Sulfate Polyacrylamide Gel Electrophoresis. The amino acid composition of the protein hydrolysate was also determined and mainly consists of lysine (8.0%), leucine (7.7%), and valine (7.2%). The protein hydrolysate may find application as a bioactive hydrolysate with an antioxidant activity of 72.6 in terms of its ability to inhibit free radicals 2,2-diphenyl-2-picryl hydrazyl with IC_50_ of 0.84%.

## 1 Introduction

Protein hydrolysate is a complex mixture of oligopeptides, peptides, and free amino acids produced from partial or extensive protein hydrolysis process (Clare and Swaizgood, 2000). The global protein hydrolysate demand is estimated to increase to 160 kilo tons in 2024 with an economic value of 800 million US dollars (Market Research MarketsandMarkets, 2018). Several types of protein hydrolysates have special biological activities (bioactive hydrolysates), such as antioxidant activity (Halim et al., 2018), antihypertensive, antimicrobial and anticoagulants and so on, therefore they are very beneficial and needed in various fields (De Castro and Sato, 2015). The properties of protein hydrolysate depend on the molecular size, amino acid composition, and amino acids sequence that shape their structure (Putra et al., 2018).

Currently the primary use of protein hydrolysate for human needs is as a raw material in children nutrition products, toiletries, energy drinks, and supplements (Clemente et al., 1999). Protein hydrolysate is also used for other purposes such as mixture of growth mediums in biotechnology-based industries (Pasupuleti and Demain, 2010) and as a high-nutritious animal feed mixture (Martínez-Alvarez et al., 2015). The main substrate used in the production of protein hydrolysates is still dominated by various types of animal protein sources such as beef, fish, milk, and eggs, as well as plant sources like wheat and soybeans (Hou et al., 2017). Animal and plant protein sources that are used as a substrate in the protein hydrolysates production are also used in human dietary needs, which may cause a competition in resources (Pasupuleti and Demain, 2010).

Black soldier fly (BSF) belongs to the order of diptera that is not a pathogenic vector for both human and livestock (Oliveira et al., 2015). Black soldier fly larvae (BSFL) is known for its ability to convert organic waste into protein and lipid rich biomass (Müller et al., 2017; Abduh et al., 2018). BSFL is also utilized as a natural controller of house fly population (*M. domestica*) and flies that are harmful to humans (Sheppard, 1983), biomarkers for forensic entomology (Pujol- Luz et al., 2008), animal and fish feed (Magalhães et al., 2017), biological agents in livestock waste management (Beskin et al., 2018), and being used as a biodiesel substrate (Nguyen et al., 2018). BSFL has the potential to be further developed as high-value bioproducts, such as protein hydrolysates. Recent works that report systematic studies on the production of protein hydrolysate from BSFL are still very scarce. Hence, this study aims to investigate the potential valorization of BSFL as a substrate to produce protein hydrolysate.

## 2 Materials and methods

### 2.1 Materials

Dried BSFL used in this study was obtained from a local BSFL producer, Biomagg® (Depok, Indonesia). The BSFL was fed with residual mixture, vegetable, and bread crumbs. N-hexane was obtained from PT Brataco (Indonesia), bromelain enzyme powder was obtained from PT Bromelain Enzim Indonesia, O-pthyaldyaldehyde was obtained from Sigma Aldrich (Singapore), HCl, NaOH, Na_2_HPO_4_, KH_2_PO_4_, Commassie Blue - R, 96% ethanol, distill water, Dithiotheritol (DTT) 99%, and sodium dodecyl sulfate (SDS) were obtained from the chemicals warehouse of School of Life Science and Technology ITB (Bandung, Indonesia).

### 2.2 Proximate analysis of BSFL

Biomass composition of BSFL was determined by proximate analysis out at the Integrated Chemical Laboratory of IPB, Bogor, Indonesia. Determination of total crude protein content was carried out using a standard Kjeldahl method with a total crude protein content calculated as %N and a conversion factor of 6.25. Determination of total crude lipid was carried out using a standard soxhlet extraction method (AOAC, 2012). Determination of total water and ash content was carried out using standard gravimetric method (AOAC, 2012). Determination of total crude carbohydrate content was obtained from the remaining percentage of another biomass component based on Eq. (1). All parameters for determining the proximate content are calculated based on percentage (%) of weight per weight (w/w).

(1) Carbohydrates (%) = (Total biomass) % - (Protein + Lipid + Ash + Water) %

### 2.3 Defatting process of BSFL

Separation of lipid component from BSFL biomass was carried out using a Soxhlet extraction method (Abduh et al., 2016). N-hexane was used as a solvent with a ratio of 1: 4 of biomass to solvent. The extraction process was carried out for 6 hours at a temperature of 70 °C. The mixture of oil and solvent was separated using a rotary evaporator for 2 hours at a temperature of 60 °C until the n-hexane solvent was completely separated and a pure oil fraction was obtained. The yield (% w/w) of oil was determined based on Eq. (2).

(2) Oil yield (% w/w) = (weight of oil (g)) / (initial sample weight (g)) × 100%

### 2.4 Protein hydrolysis of BSFL

Protein hydrolysis of defatted BSFL was carried out using a bromelain enzyme with an activity of 200 CDU/mg according to the procedure suggested by Auwal et al. (2017). A total of 10 grams defatted BSFL was dissolved in 50 ml phosphate buffer solution at different pH and the enzyme was added at different concentrations in a 250 ml erlenmeyer flask. The hydrolysis reaction was carried out in a water bath shaker at a temperature of 50 °C, rotation speed of 150 rpm, and at different time variations according to the conditions in Table 1. The hydrolysis reaction was terminated by heating the sample at a temperature of 90 °C for 10 minutes using a water bath. Remaining BSFL samples were completely hydrolyzed by using 6 M HCl at a temperature of 110 °C for 24 hours to determine the total free amino acid content.

**Table 1:** Factors and level for the hydrolysis of defatted BSFL using a face-centered CCD.

Factors	Level		
	-1	0	1
Enzyme concentration, E/S (%)	1	3	5
pH	6	7	8
time, t (hours)	3	13,5	24

The mixture of protein hydrolysate from BSFL was separated by centrifugation as suggested by Caligiani et al. (2018). Briefly, each sample was poured into a 15 ml centrifuge tube and centrifuged at 4000 rpm for 30 minutes at 25 °C. The supernatant was separated from the precipitated part and stored in a 20 ml bottle which was tightly closed for further processing. The supernatant obtained was stored in a refrigerator at a temperature of 4 °C for further analysis. Protein hydrolysate from BSFL was dried using a freeze-drying method. Briefly, 35 ml of the supernatant of protein hydrolysate from BSFL was frozen in the freezer until the phase changed into a solid. The solid sample was then placed inside a freeze drier at temperature and pressure of -55 °C and 1500 mTorr, respectively until a constant weight was obtained. Dried protein hydrolysate from BSFL was stored in a refrigerator at a temperature of 4 °C for further analysis.

### 2.5 Design of experiments and optimization

Optimization condition of protein hydrolysis was carried out using Minitab 8.0 with a face-centered Central Composite Design (CCD) with the factors and level for the hydrolysis of defatted BSFL are shown in Table 1. The data were modelled using a second-order polynomial as shown in equation Eq. (3).

(3) $$y=b_{0}+\overset{3}{\underset{i=1}{\sum }}b_{i}X_{i}+\;\overset{3}{\underset{i\;=1}{\sum }}b_{i}X_{i}^{2}+\overset{3}{\underset{i<j=2}{\sum }}b_{ij}X_{i}X_{j},$$

where y is degree of hydrolysis (%), b_0_ is a constant value, b_i_ is coefficient of each variables, X_1_ is enzyme concentration (%), X_2_ is pH, and X_3_ is hydrolysis time (hours). The regression equation was obtained by backward elimination of non-significant coefficients as suggested by Abduh et al. (2016). The optimum conditions for the hydrolysis defatted biomass of BSFL were determined using the numerical optimization function available in Minitab 8.0.

### 2.6 Determination of yield and productivity of protein hydrolysate

The yield and productivity of protein hydrolysates in the form of freeze dried powder were calculated using Eq. (4) and Eq. (5), respectively based on the following assumptions:

• Yield was calculated based on the initial weight of hydrolysis substrate, the defatted dried BSFL
• Productivity was calculated for 1 batch of production processes that is comparable with a duration of about 2 days.
• The reaction volume used during the production process of the BSFL protein was 60 ml
• The oil and solvent mixture were completely separated; hence the amount of remaining solvent was calculated as the difference from the initial solvent and the oil produced.

(4) Yield (%) = m_final_ (g) / m_initial_ (g) × 100%

where m_final_ is mass of freeze dried protein hydrolysate from BSFL (gram) and m_initial_ is mass of initial defatted BSFL sample.

(5) Productivity (g/L/batch) = m_final_ (g) / (V_reaction_ (mL) × 1 batch)

where m_final_ is mass of freeze dried protein hydrolysate and V_reaction_ is volume of hydrolysis reaction in Erlenmeyer flask.

### 2.7 Determination of protein concentration

Protein concentration of protein hydrolysate from BSFL was determined using a Bradford method (Kruger, 2009). Briefly, 0.1 ml of the protein hydrolysate from BSFL was poured into a cuvet. Each sample was added with 5.0 ml of commassie brilliant blue-G solution. The mixture was incubated for approximately 2 minutes at 25 °C. The sample absorbance was determined using a Shimadzu UV-3101 spectrophotometer instrument at a wavelength of 595 nm. Protein concentration was calculated was based on a BSA standard curve.

### 2.8 Determination of degree of hydrolysis

Degree of hydrolysis (DH) for protein hydrolysate from BSFL was determined based on the method of Nielsen et al. (2010) and Mirzaei et al. (2015) with slight modifications. O-pthyaldyaldehyde (OPA) reagent was always freshly made before used to determine the DH of the sample. Briefly, 7.62 g of sodium tetraborate decahydrate and 20 mg SDS were dissolved in 150 ml of deion water and stirred using a magnetic stirrer until a homogeneous solution was obtained. The mixture was added with 160 mg of 97% O-pthyaldyaldehyde powder which was dissolved in 4 ml of 95% ethanol. The mixture was added with 176 mg 99% of DTT and stirred until homogeneous. The final volume of the OPA reagent mixture was made up to 200 mL by adding deion water. OPA reagent was stored in a dark bottle at 25 °C.

The analysis of DH was carried out by adding 0.4 mL of a protein hydrolysate sample containing 200–300 ug/ml of protein with 3.0 mL of OPA reagent to determine the amount of free amino acid in the solution. L-serine amino acid (0.01–0.10 mg/ml) was used as a standard solution to make a standard curve. The standard solution and all samples were incubated at 25 °C for 2 minutes. The standard solution and all samples were measured for absorption at a wavelength of 340 nm using a Shimadzu UV-3101 spectrophotometer. Absorption of each sample was converted using the standard linear curve regression equation obtained. The degree of hydrolysis was calculated based on Eq. (6).

(6) DH (%) = h1 / h0 × 100%

where DH is Degree of Hydrolysis (%), h_1_ is peptide concentration, and h_0_is total amino acid (counted as L-serine).

### 2.9 Determination of amino acid

The composition of amino acid in protein hydrolysate from BSFL was analyzed using a High-Performance Liquid Chromatography (HPLC) at the Integrated Chemical Laboratory of Bogor Agricultural University, Indonesia.

### 2.10 Determination of molecular weight distribution

Molecular weight distribution of protein hydrolysate from BSFL was determined using a Sodium Dodecyl Sulfate Poly Acrylamide-Gel Electrophoresis (SDS-PAGE) method (Hall et al., 2017). Polyacrylamide gel for electrophoresis was made by mixing a solution of acrylamide bis-acrylamide mix 12%, deion water, Tris-Cl buffer 0.5 M pH 6.8 (staking gel), Tris-Cl buffer 1.5 M pH 8.8 (separating gel), SDS, ammonium persulfat, and Temed, respectively. Protein samples and protein hydrolysates were prepared by mixing 50 μL samples with a staining solution containing 2-mercaptoethanol, 2% SDS, and 0.05% bromophenol blue in 62.5 mM Tris/HCl buffer (pH 6.8). The samples were incubated at 90 °C for 10 minutes in a water bath. Electrophoresis was operated using a Biorad SDS-PAGE device at a voltage of 100 V, current of 0.1 A, for 150 minutes at 25 °C. A ladder protein of 14–116 kDa was used as a comparison to determine the distribution of protein molecular weight. The electrophoresis gel was stained using a staining solution consisting of commassie brilliant blue R-250, 40% technical grade methanol in a volume basis (v/v), 10% v/v glacial acetic acid, and 50% v/v deion water. The gel coloring process with staining solution was carried out for 12 hours. The colored gel was rinsed and soaked in a destaining solution consist of 10% v/v methanol in deion water. The destaining process was carried out for 12 hours until the protein band in the gel could be clearly seen.

### 2.11 Determination of antioxidant activity

Antioxidant activity of protein hydrolysate from BSFL was determined based on the scavenging ability of DPPH (2,2-diphenyl-1-picrylhydrazyl) free radicals expressed in the form of IC_50_ concentrations (Zielińska et al., 2017). DPPH free radical inhibition activity analysis was carried out at the Chemigreecal Laboratory of the Padjadjaran University (UNPAD), Jatinangor. Briefly, DPPH free radicals were dissolved in methanol and added with samples at several concentrations (0.25–1.25% v/v). All samples were incubated for 30 minutes in a dark room and then tested for absorption using a spectrophotometer at a wavelength of 517 nm. The value of free radical inhibition was determined using Eq. (7). The measurement was carried out at least twice and the average values were reported.

(7) Free radical inhibition activity (%) = (A_control_- A_sampel_) / A_control_ × 100%

where A_control_ is the absorbance of control sample at λ = 517nm, and A_sample_ is the absorbance of the sample at λ = 517 nm.

## 3 Results and discussion

### 3.1 Biomass composition of BSFL

Biomass composition of BSFL was analyzed using a proximate analysis method to determine crude protein, lipid, carbohydrate, fiber, and ash content. BSFL has crude protein and lipid content of 25.6% w/w and 35.5% w/w, respectively. The content of protein, lipid, carbohydrates, fiber, and ash in BSFL depends on the substrate consumed (Müller et al., 2017). Liu et al. (2017) cultivated BSFL using a commercial chicken feed substrate and produced BSFL biomass with a protein content up to 39.2% w/w. Abduh et al. (2018) reported that BSFL has a protein content of 45% w/w when fed with defatted residual Philippine tung seeds (*Reutalis trisperma*) during its cultivation period. The BSFL used in this study has a lower protein content compared to the other studies. It is probably due to the substrate composition used in this study that comprises a mixture of vegetable and plant-based food residues. However, the protein content is still greater than 25% w/w of BSFL used in this study is still classified as a high protein biomass (Mulder et al., 2016).

**Fig. 1:**
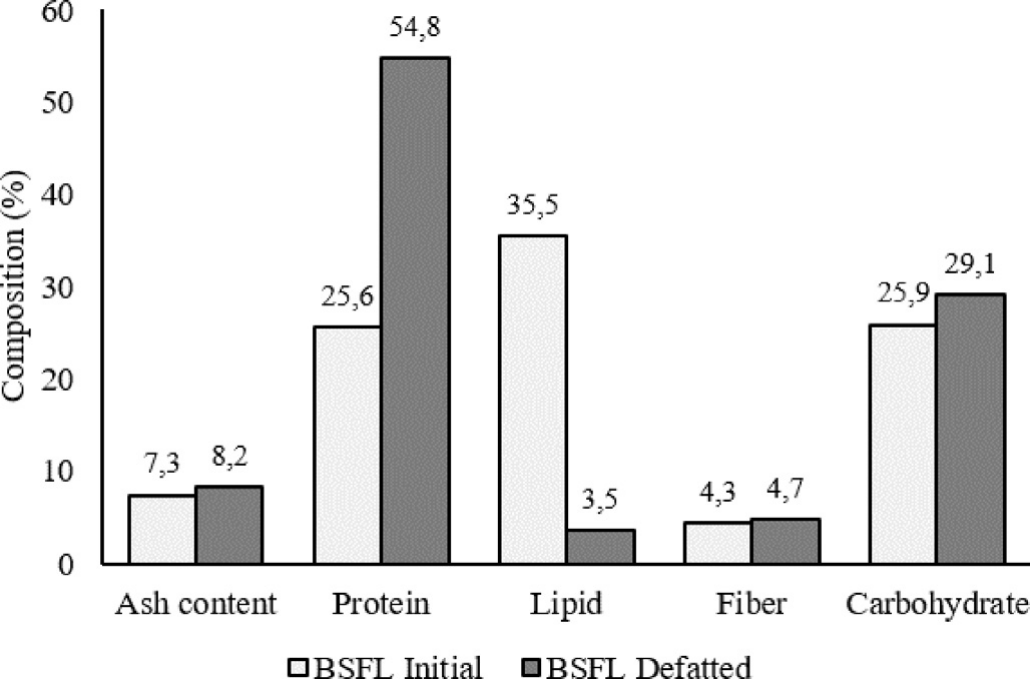
Composition of initial BSFL biomass and defatted BSFL.

The relatively high lipid content in the BSFL (35% w/w) was separated prior to the hydrolysis process was. The lipid component was separated by a Soxhlet extraction method using an n-hexane as a solvent. After the lipid component has been removed, the BSFL protein fraction was doubled to 54, 81% as compared to the initial content (25.6%) as shown in Fig. 1. Increased protein content after lipid extraction process was also reported by Longvah et al. (2011) with a 50% increased of protein content for silkworm prepupae. So that, the hydrolysis process using bromelain enzyme was more effective after the separation of lipid content in BSFL because the non-protein component have been reduced (Coulen et al., 2017).

### 3.2 Yield and productivity of protein hydrolysate from BSFL

**Fig. 2:**
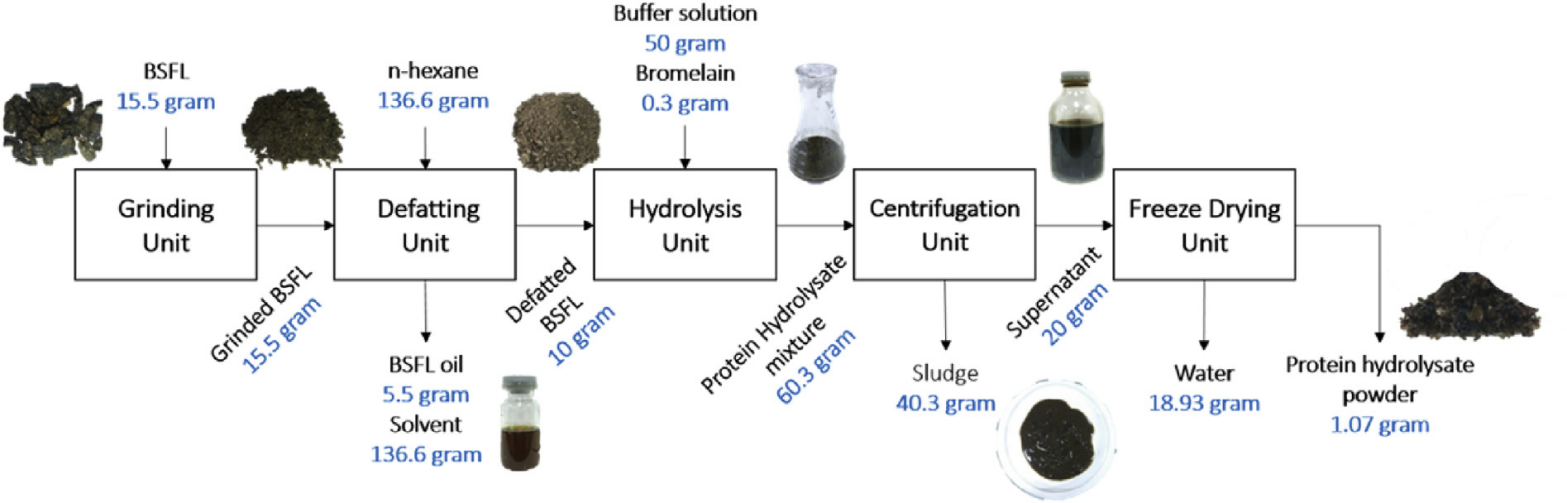
Schematic diagram to produce protein hydrolysate from BSFL.

The yield and productivity of protein hydrolysate from BSFL in the form of freeze dried solid was calculated to investigate it's potential to be developed on a larger scale production. As much as 1.07 grams of freeze dried protein hydrolysates from BSFL were obtained from a series of production processes (Fig. 2). The yield of freeze dried protein hydrolysates from BSFL reached up to 10.7% w/w and 6.9% w/w based on the defatted BSFL and based on initial non-defatted BSFL, respectively. Similar findings were reported by Hall et al. (2017) which produced solid protein hydrolysates from cricket biomass with yields ranging from 5.2 to 11.7% w/w. The yield of protein hydrolysates varies depending on the hydrolysis conditions performed (Hall et al., 2017). Based on the experiments conducted in this study, the productivity of protein hydrolysates from BSFL reached up to 21 mg/ml/batch.

### 3.3 Effect of enzyme concentration, pH and time on protein concentration of protein hydrolysate from BSFL

Protein concentration of the protein hydrolysate from BSFL was analyzed using a Bradford method. Protein concentration analysis was carried out to determine the range of dissolved protein concentration in the protein hydrolysate which can be used as a reference for determining the degree of hydrolysis using the OPA method. The results of protein hydrolysate concentration obtained from experimental data and regression model are shown in Table.2. In general, protein concentration of protein hydrolysate from BSFL varies in the range 240–310 μg/mL. The highest protein concentration of protein hydrolysate from BSFL (308.7 μg/mL) were obtained at 3% enzyme concentration, pH 8, and 13.5 hours of hydrolysis time. The lowest concentration (249.1 μg/ml) was obtained under the conditions at 1% enzyme concentration, pH 6, and 24 hours of hydrolysis time. The varying protein concentrations in the protein hydrolysate from BSFL showed that protein concentration was influenced by enzyme concentration, pH and hydrolysis time.

**Table 2:** Protein concentration and DH for protein hydrolysate from BSFL of different condition of enzyme, pH, and hydrolysis time'.

Running	Reaction Condition			Protein Concentration (μg/mL)		Degree of Hydrolysis (%)	
	Enzyme (%)	pH	Time (hours)	Experiment	Model	Experiment	Model
1	3	7	24	244.4	265.1	37.7	35.0
2	3	7	13.5	307.3	299.6	23.4	23.5
3	5	6	3	247.7	253.8	26.5	28.6
4	3	7	13.5	307.3	299.6	23.4	23.5
5	5	7	13.5	293.9	295.5	10.6	16.5
6	3	7	13.5	323.4	299.6	20.9	23.5
7	5	6	24	256.8	250.5	31.3	32.3
8	1	8	3	290.6	287.7	14.6	14.0
9	1	7	13.5	268.2	292.4	19.6	12.3
10	5	8	3	286.8	281.3	20.2	14.3
11	1	6	24	241.5	237.9	17.9	24.3
12	3	6	13.5	282.0	289.7	43.7	31.1
13	3	7	13.5	307.3	299.6	23.4	23.5
14	5	8	24	300.6	299.6	41.2	38.3
15	3	7	3	263.4	268.5	19.7	21.0
16	3	7	13.5	307.3	299.6	23.4	23.5
17	3	8	13.5	308.7	326.7	19.6	30.8
18	3	7	13.5	307.3	299.6	23.4	23.5
19	1	8	24	299.6	284.4	39.9	38.1
20	1	6	3	271.0	262.9	17.3	20.5

### 3.4 Effect of enzyme concentration, pH, and time on the DH of protein hydrolysate from BSFL

**Fig. 3:**
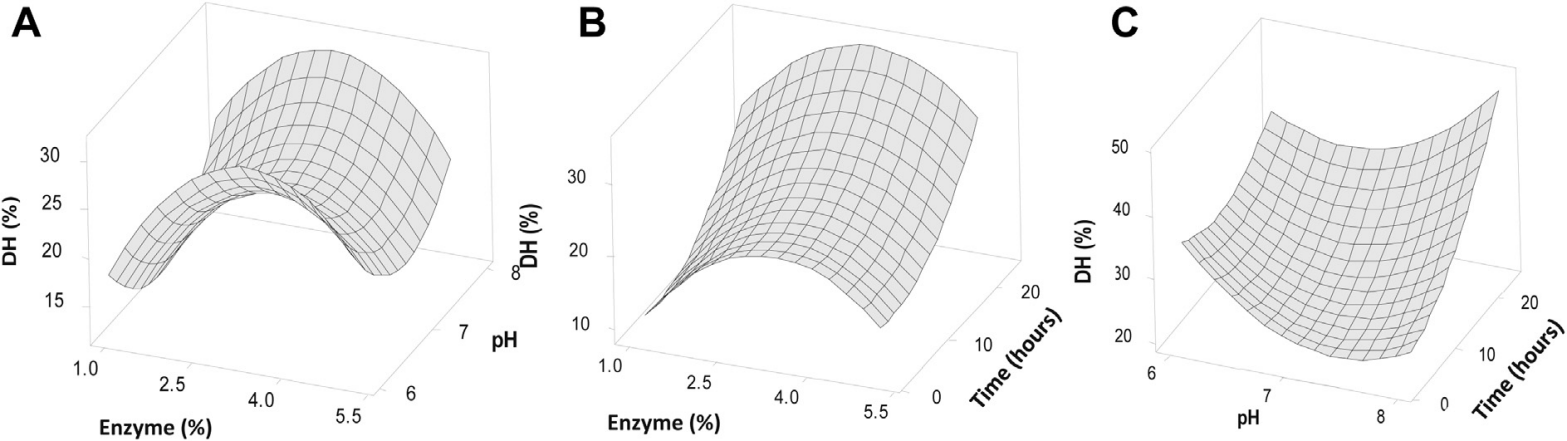
Surface response from the interaction between variables on the DH with respect to enzyme concentration and pH (A), enzyme concentration and time (B), and pH and time (C).

The DH of the protein hydrolysates from BSFL was determined using the OPA method which calculated the ratio of free L-serine amino acids in the sample and total amino acid from extensive hydrolysis. The DH values of the protein hydrolysate from BSFL are shown in Table 2 and the coefficients of the regression equation are shown in Table 3. The profile of enzyme concentration, pH, and hydrolysis time effect on DH is shown in Fig. 3.

**Table 3:** Regression model coefficient for predicting of the DH of protein hydrolysate from BSFL.

Variable	Coefficient
Constant	389
X_1_	21.6
X_2_	-107.9
X_3_	-3.83
X_1_^2^	-2.28
X_2_^2^	7.44
X_3_^2^	0.04
X_1_X_2_	-0.98
X_1_X_3_	-0.001
X_2_X_3_	0.49

X_1_: enzyme concentration (%), X_2_: pH, and X_3_: time (hour).

Protein hydrolysate from BSFL was successfully obtained from a series of production processes, with the DH values varies in the range of 10–44%. This finding shows that the DH value is influenced by various factors, including the concentration of enzymes, pH, and hydrolysis time (Pasupuleti and Demain, 2010). Hydrolysis conditions of 3% enzyme concentration, pH 6, and hydrolysis time of 13.5 hours had the highest DH value, reaching up to 43.7%. Meanwhile the hydrolysis conditions at an enzyme concentration of 5%, pH 7 and the hydrolysis time of 13.5 hours had the lowest DH value (10.6%).

The optimum conditions for the hydrolysis of protein in BSFL according to the regression model are the enzyme concentration of 3%, pH 8, and hydrolysis time of 24 hour with a DH up to 47.4%. An enzyme concentration of 3% produces the highest DH compared to enzyme concentrations of 1% and 5%. A maximum DH value at 3% enzyme concentration was also reported for the hydrolysis of cricket proteins (*Gryllodes sigillatus*) using alcalase, with a DH up to 52% (Hall et al., 2017). Likewise, protein hydrolysates from Chinese sturgeon (*Acipenser sinensis*) had the highest DH value which when the hydrolysis was carried out using a 3% concentration of papain enzyme (Noman et al., 2018). As such occurred because the interaction of protein substrate with bromelain had reached its maximum saturation (plateaued state) and the hydrolysis process will not run effectively and efficiently when the enzyme concentration was greater than 3% (Aspmo et al., 2005).

### 3.5 Molecular weight distribution of protein hydrolysate from BSFL

**Fig. 4:**
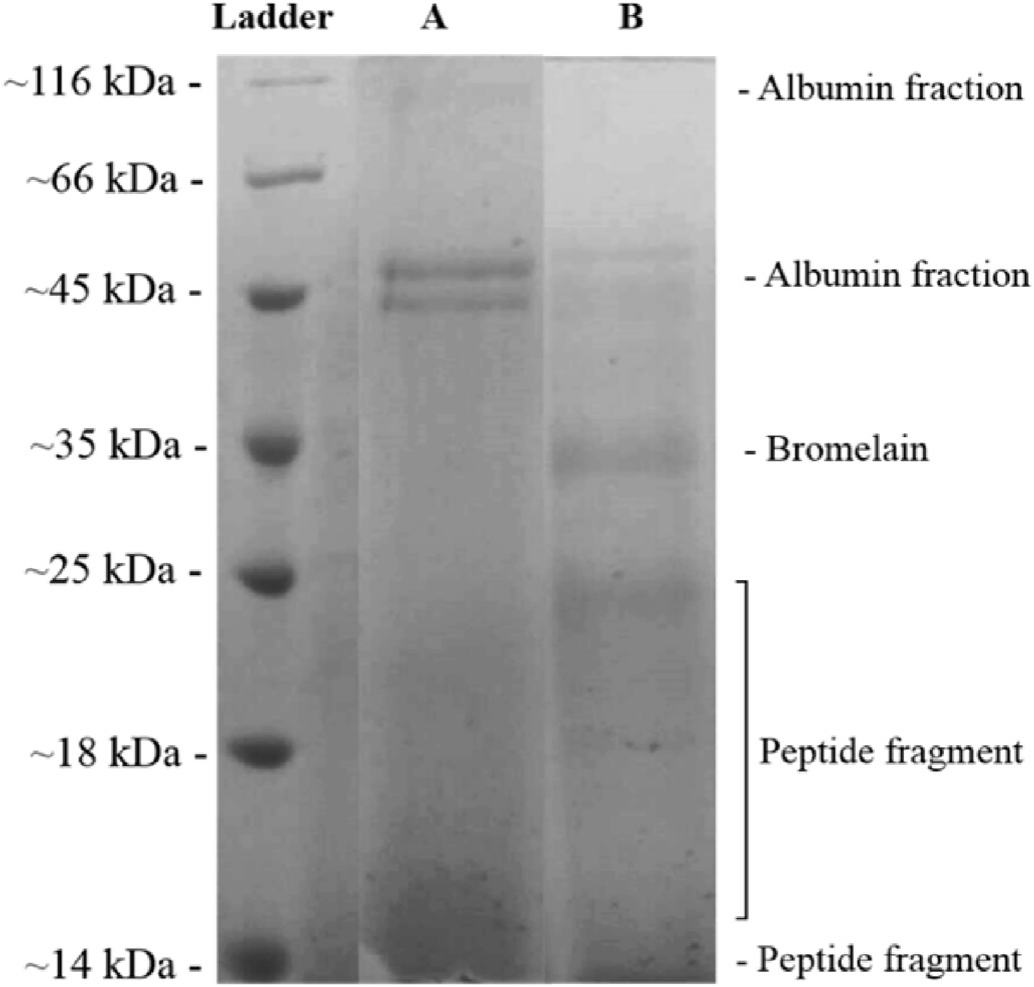
Distribution of molecular weight of protein extract sample without enzyme (A), and protein hydrolysate from BSFL (enzyme concentration of 3%, pH 7, and hydrolysis time of 13.5 hours) (B).

Molecular weight distribution of protein sample without the addition of enzymes and protein hydrolysate from BSFL was compared using the SDS-PAGE method. The results of the electrophoresis and ladder protein as a comparison are shown in Fig. 4. In general, protein bands and peptides in protein water extract or control sample (A) appear in a molecular size ranges of around 14–116 kDa. Meanwhile the protein hydrolysate (B) sample has bands in a molecular size range of about 14–45 kDa.

Protein bands appeared around 116 kDa and 45 kDa were possibly albumin fraction, because this protein fraction are hydrophilic and also water soluble (Caligiani et al., 2018). Protein hydrolysate from BSFL (A) also has a 45 kDa band, but had a thinner appearance as compared to the control sample (B). There is another difference between the control sample (A) and (B), which is the presence of protein band at a molecular weight around 35 kDa that belongs to bromelain. Bromelain itself is a protease with a molecular weight varying from 28 - 33 kDa (Murachi, 1964; Ketnawa et al., 2012). This result occurred because bromelain was added to protein hydrolysate from BSFL as a protease to catalyze the hydrolysis process while bromelain was not added to the control sample without hydrolysis treatment.

### 3.6 Composition of amino acid in the protein hydrolysate from BSFL

Amino acid composition of protein hydrolysate from BSFL was analyzed using HPLC instruments and the results are shown in Table 4. The most dominant amino acid in the protein hydrolysate from BSFL was glutamic acid (18.4%). Janssen et al. (2017) and Müller et al. (2017) reported that amino acids in BSFL were dominated by glutamic acid. Likewise, in the prepupa of BSF sample, the most dominant amino acid component is also glutamic acid (Surendra et al., 2016). Protein hydrolysate from another insect species such as tropical banded crickets (*Gryllodes sigillatus*) also has glutamic acid as the most dominant amino acid with a composition up to 19% (Hall et al., 2017).

**Table 4:** Amino acid composition of protein hydrolysate from BSFL.

Amino acids	This study (Protein hydrolysate from BSFL)	Müller et al. (2017) (BSFL)	Janssen et al., 2017 (BSFL)	Surendra et al. (2016) (BSFL Prepupa)	Hall et al. (2017) (Protein hydrolysate from *G. sigillatus*)
Alanine	12.1	6.2	4.7	7.9	16.6
Arginine*	3.3	6.2	4.6	6.6	18.1
Aspartic acid	9.7	10.3	12.6	7.8	0.8
Glutamic acid	18.1	12.2	12.1	8.4	19.9
Phenylalanine*	4.2	6.2	7.2	4.4	2.6
Glycine	6.1	5.4	3.9	7.3	3.7
Histidine*	2.7	4.8	3.6	5.0	3.4
Isoleucine*	5.3	4.8	5.8	4.5	2.6
Lysine*	8.0	7.4	9.2	6.5	3.6
Leucine*	7.7	7.7	8.0	6.9	8.4
Methionine*	1.7	0.6	2.5	2.6	0.6
Proline	-	6.2	4.34	6.2	3.3
Serine	4.5	4.1	4.0	4.5	6.7
Cysteine	-	0.5	1.33	3.3	3.6
Tyrosine	4.3	6.0	6.3	7.0	1.4
Threonine*	5.0	4.5	4.9	4.4	2.6
Tryptophan*	-	-	-	-	0.7
Valine*	7.3	6.7	5.6	7.2	1.4

* Essential amino acid group.

In this study, protein hydrolysate from BSFL also contains a relatively high amount of alanine (12.1%). This is because the bromelain enzyme used in hydrolysis process of BSFL mostly consisted of L-alanine amino acid (Murachi, 1964). The protein hydrolysate from BSFL contains almost all essential amino acid. Hydrophobic amino acids, such as, lysine (8.0%), leucine (7.7%) and valine (7.3%) were the most dominant essential amino acids. High amount of hydrophobic amino acid in a protein hydrolysate, can be a sign of the ability to scavenge or inhibit free radicals (Saadi et al., 2015).

### 3.7 Antioxidant activity of protein hydrolysate from BSFL

**Fig. 5:**
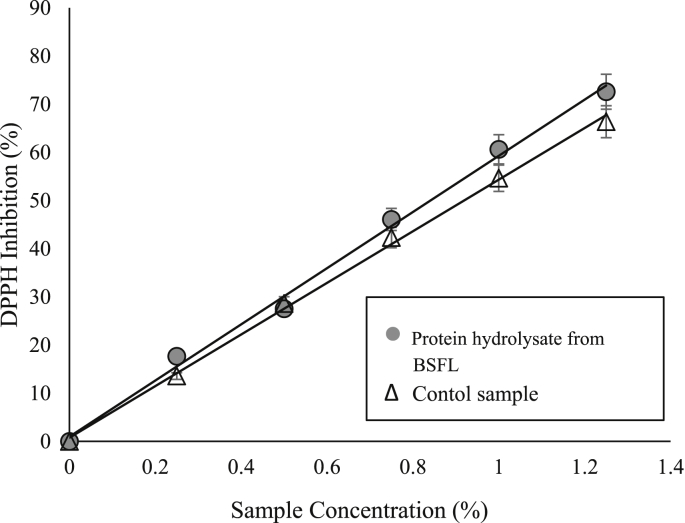
DPPH free radical inhibition profile of protein hydrolysates from BSFL and control at various concentrations (0–1.25% v/v).

According to the DPPH free radical scavenging analysis, both protein hydrolysate from BSFL and control sample had the ability to inhibit DPPH free radical up to 72.6 ± 0.41% v/v and 66.3 ± 0.40% v/v, respectively at a concentration of 1.25% v/v (Fig. 5). The activity of DPPH free radical inhibition is also found in the protein hydrolysate from sea cucumber or stone fish (*Actynopiga lecanora*) which had an inhibitory ability up to 50% at 1,0% v/v sample concentration (Auwal et al., 2017). In addition, Zielińska et al. (2017) also reported that protein hydrolysates in three types of insects had DPPH* free radical inhibitory activity indicated by IC_50_ values. IC_50_ values of protein hydrolysate from BSFL and control samples were 0.84 ± 0,001% v/v and 0.92 ± 0,015% v/v, respectively. It means that protein hydrolysate from BSFL has a higher DPPH free radical inhibition activity then protein hydrolysate from sea cucumber (IC_50_ ∼ 1% v/v) reported by Auwal dkk., (2017). In another study, Zielińska et al. (2017) reported that protein hydrolysate from selected insect, particularly *Schistocerca gregaria, Gryllodes sigillatus*, and larva of *Tenobrio mollitor* have an IC_50_ up to 0.004–0.010% v/v which were better than the results obtained in this study.

The antioxidant activity of a protein hydrolysate is the result of various synergistic mechanisms, including the deterrence or inhibition of free radicals, inhibition of lipid peroxidation processes, metal ionization, countering oxidative reactions by oxygen-containing compounds, and electron transfer processes (Chen et al., 1996). The DPPH free radical inhibition activity possessed by protein hydrolysate from BSFL is influenced by the amino acid composition in it. Aromatic amino acids such as phenylalanine, and tyrosine are able to stabilize DPPH free radicals through the mechanism of proton donors to electron-deficient free radicals (Dávalos et al., 2004). The proton donor process results in propagating termination of free radical reaction (Auwal et al., 2017). In addition, amino acids that have hydrophobic properties played an important role in the process of deterring free radicals (Saadi et al., 2015). This occurs because of the ability of hydrophobic amino acids to increase access of free radicals to related amino acids, thereby reducing access of free radicals in attacking compounds and targeted cells (Nasri, 2017). Thus, protein hydrolysate from BSFL has the potential as bioactive hydrolysate with an antioxidant activity based on the amino acid composition and the ability to inhibit DPPH free radicals.

## 4 Conclusions

Protein hydrolysate from defatted BSFL that contains 50 % w/w of protein had been successfully synthesized using a bromelain enzyme as a biocatalyst. The DH of protein hydrolysate from BSFL lies in the range of 10–48%. The optimum conditions of the hydrolysis process with a DH of 47.4%, occurred at 3% of enzyme concentration, pH 8, and hydrolysis time of 24 hours as suggested by regression model. The protein hydrolysate from BSFL had a yield of 10.7% w/w and a productivity of 21 mg/mL/batch. The protein hydrolysate from BSFL contains high amount of hydrophobic essential amino acid, particularly lysine (8.0%), leucine (7.7%), and valine (7.3%). The protein hydrolysate from BSFL had an antioxidant activity in terms of its ability to scavenge DPPH free radicals up to 77% at 1.25% sample concentration with IC_50_ value of 0.84% v/v.

## Declarations

### Author contribution statement

Mochamad Firmansyah: Conceived and designed the experiments; Performed the experiments; Analyzed and interpreted the data; Wrote the paper.

Muhammad Yusuf Abduh: Conceived and designed the experiments; Analyzed and interpreted the data; Contributed reagents, materials, analysis tools or data; Wrote the paper.

### Funding statement

This work was supported by a research grant from the Ministry of Research, Technology, and Higher Education Indonesia (PN-1-23-2018).

### Competing interest statement

The authors declare no conflict of interest.

### Additional information

No additional information is available for this paper.

## References

[bib1] Abduh M.Y., Rasrendra C.B., Subroto E., Manurung R., Heeres H.J. (2016). Experimental modelling studies on the solvent assisted hydraulic pressing of dehulled rubber seeds. Ind. Crops Prod..

[bib2] Abduh M.Y., Nadia M.H., Manurung R., Putra R.E. (2018). Factors affecting the bioconversion of Philippine tung seed by black soldier fly larvae for the production of protein oil-rich biomass. J. Asia Pac. Entomol..

[bib40] AOAC (2012). Official Methods of Analysis of AOAC International.

[bib3] Aspmo S.I., Horn S.J., Eijsink V.G. (2005). Enzymatic hydrolysis of Atlantic cod (*Gadus morhua* L.) viscera. Process Biochem..

[bib4] Auwal S.M., Zarei M., Abdul-Hamid A., Saari N. (2017). Response surface optimisation for the production of antioxidant hydrolysates from stone fish protein using bromelain. Evid. Based Complement Alternat. Med..

[bib5] Beskin K.V., Holcomb C.D., Cammack J.A., Crippen T.L., Knap A.H., Sweet S.T., Tomberlin J.K. (2018). Larval digestion of different manure types by the black soldier fly (Diptera: stratiomyidae) impacts associated volatile emissions. Waste Manag..

[bib6] Caligiani A., Marseglia A., Leni G., Baldassarre S., Maistrello L., Dossena A., Sforza S. (2018). Composition of black soldier fly prepupae systematic approaches for extraction fractionation of proteins, lipids and chitin. Food Res. Int..

[bib7] Chen H.M., Muramoto K., Yamauchi F., Nokihara K. (1996). Antioxidant activity of designed peptides based on the antioxidative peptide isolated from digests of a soybean protein. J. Agric. Food Chem..

[bib8] Clare D.A., Swaisgood H.E. (2000). Bioactive milk peptides: a prospectus. Int. J. Dairy Sci..

[bib9] Clemente A., Vioque J., Sánchez-Vioque R., Pedroche J., Bautista J., Millán F. (1999). Protein quality of chickpea (*Cicer arietinum* L.) protein hydrolysates. Food Chem..

[bib10] Coulen S.C., Sanders J.P., Bruins M.E. (2017). Valorisation of proteins from rubber tree. Waste Biomass Valorization.

[bib11] Dávalos A., Miguel M., Bartolome B., Lopez-Fandino R. (2004). Antioxidant activity of peptides derived from egg white proteins by enzymatic hydrolysis. J. Food Prot..

[bib12] De Castro R.J.S., Sato H.H. (2015). A response surface approach on optimization of hydrolysis parameters for the production of egg white protein hydrolysates with antioxidant activities Biocatal. Agric. Biotechnol..

[bib14] Halim N.R.A., Azlan A., Yusof H.M., Sarbon N.M. (2018). Antioxidant anticancer activities of enzymatic eel (*Monopterus sp*) protein hydrolysate as influenced by different molecular weight. Biocatal. Agric. Biotechnol..

[bib15] Hall F.G., Jones O.G., O'Haire M.E., Liceaga A.M. (2017). Functional properties of tropical banded cricket (*Gryllodes sigillatus*) protein hydrolysates. Food Chem..

[bib16] Hou Y., Wu Z., Dai Z., Wang G., Wu G. (2017). Protein hydrolysates in animal nutrition: industrial production, bioactive peptides, functional significance. J. Anim. Sci. Biotechnol..

[bib17] Janssen R.H., Vincken J.P., van den Broek L.A., Fogliano V., Lakemond C.M. (2017). Nitrogen-to-Protein conversion factors for three edible insects: *Tenebrio molitor, Alphitobius di*aperinus, Hermetia illucens. J. Agric. Food Chem..

[bib18] Ketnawa S., Chaiwut P., Rawdkuen S. (2012). Pineapple wastes: a potential source for bromelain extraction. Food Bioprod. Process..

[bib41] Kruger N.J. (2009). The Bradford method for protein quantitation. The Protein Protocols Handbook.

[bib20] Liu X., Chen X., Wang H., Yang Q., ur Rehman K., Li W., Cai M., Li Q., Mazza L., Zhang J., Yu Z. (2017). Dynamic changes of nutrient composition throughout the entire life cycle of black soldier fly. PLoS One.

[bib21] Longvah T., Mangthya K., Ramulu P. (2011). Nutrient composition protein quality evaluation of eri silkworm (*Samia ricinii*) prepupae pupae. Food Chem..

[bib22] Marketsandmarket. 2018.https://www.marketresearch.com/MarketsandMarkets-v3719/Protein-Hydrolysates-Type-Milk-Meat-11745269/, accessed on 1 September 2018.

[bib23] Magalhães R., Sánchez-López A., Leal R.S., Martínez-Llorens S., Oliva-Teles A., Peres H. (2017). Black soldier fly (*Hermetia illucens*) pre-pupae meal as a fish meal replacement in diets for European seabass (*Dicentrarchus labrax*). Aquaculture.

[bib24] Martínez-Alvarez O., Chamorro S., Brenes A. (2015). Protein hydrolysates from animal processing by-products as a source of bioactive molecules with interest in animal feeding: a review. Food Res. Int..

[bib42] Mirzaei M., Mirdamadi S., Ehsani M.R., Aminlari M., Hosseini E. (2015). Purification and identification of antioxidant and ACE-inhibitory peptide from Saccharomyces cerevisiae protein hydrolysate. J Funct. Foods.

[bib43] Mulder W., van der Peet-Schwering C., Hua N.P., van Ree R. (2016). Proteins for Food, Feed and Biobased Applications: Biorefining of Protein Containing Biomass. IEA Bioenergy Task 42.

[bib25] Müller A., Wolf D., Gutzeit H.O. (2017). The black soldier fly, *Hermetia illucens*–a promising source for sustainable production of proteins, lipids bioactive substances. Z. Naturforsch. C Biosci..

[bib26] Murachi T. (1964). Amino acid composition of stem bromelain. Biochemistry.

[bib27] Nasri M. (2017). Protein hydrolysates biopeptides: production, biological activities, applications in foods health benefits. A review. Adv. Food Nutr. Res..

[bib28] Nguyen H.C., Liang S.H., Chen S.S., Su C.H., Lin J.H., Chien C.C. (2018). Enzymatic production of biodiesel from insect fat using methyl acetate as an acyl acceptor: optimization by using response surface methodology. Energy Convers. Manag..

[bib44] Nielsen P.M., Petersen D., Dambmann C. (2010). Improved method for determining food protein degree of hydrolysis. J. Food Sci..

[bib29] Noman A., Xu Y., AL-Bukhaiti W.Q., Abed S.M., Ali A.H., Ramadhan A.H., Xia W. (2018). Influence of enzymatic hydrolysis conditions on the degree of hydrolysis functional properties of protein hydrolysate obtained from Chinese sturgeon (*Acipenser sinensis*) by using papain enzyme. Process Biochem..

[bib31] Oliveira F., Doelle K., List R., O’Reilly J.R. (2015). Assessment of Diptera: stratiomyidae, genus *Hermetia illucens* (L., 1758) using electron microscopy. J. Entomol. Zool. Stud..

[bib32] Pujol-Luz J.R., Francez P.A.D.C., Ururahy-Rodrigues A., Constantino R. (2008). The black soldier-fly, *Hermetia illucens* (Diptera, stratiomyidae), used to estimate the postmortem interval in a case in amapá state, Brazil. J. Forensic Sci..

[bib45] Pasupuleti V.K., dan Demain A.L. (2010). Protein Hydrolysates in Biotechnology.

[bib34] Putra S.N.K.M., Ishak N.H., Sarbon N.M. (2018). Preparation characterization of physicochemical properties of golden apple snail (*Pomacea canaliculata*) protein hydrolysate as affected by different proteases. Biocatal. Agri. Biotechnol..

[bib35] Saadi S., Saari N., Anwar F., Hamid A.A., Ghazali H.M. (2015). Recent advances in food biopeptides: production, biological functionalities therapeutic applications. Biotechnol. Adv..

[bib36] Sheppard C. (1983). House fly lesser fly control utilizing the black soldier fly in manure management systems for caged laying hens. Environ. Entomol*.*.

[bib37] Surendra K.C., Olivier R., Tomberlin J.K., Jha R., Khanal S.K. (2016). Bioconversion of organic wastes into biodiesel animal feed via insect farming. Renew. Energy.

[bib39] Zielińska E., Baraniak B., Karaś M. (2017). Antioxidant anti-inflammatory activities of hydrolysates peptide fractions obtained by enzymatic hydrolysis of selected heat-treated edible insects. Nutrients.

